# The self‐memory system: Exploring developmental links between self and memory across early to late childhood

**DOI:** 10.1111/cdev.14163

**Published:** 2024-09-09

**Authors:** Josephine Ross, Jacqui Hutchison, Sheila J. Cunningham

**Affiliations:** ^1^ Psychology, School of Humanities, Social Sciences and Law University of Dundee Dundee UK; ^2^ School of Psychology University of Aberdeen Aberdeen UK; ^3^ School of Social and Applied Sciences Abertay University Dundee UK

## Abstract

This study tests whether developments in self‐knowledge and autobiographical memory across early to late childhood are related. Self‐descriptions and autobiographical memory reports were collected from 379 three‐ to eleven‐year‐old predominantly white Scottish children, *M*_age_ = 90.3 months, SD = 31.1, 54% female. Episodic memory was measured in an enactment task involving recall and source monitoring of performed and witnessed actions. The volume and complexity of self‐knowledge and autobiographical memory reports increased with age, as did source monitoring ability and recall bias for own actions. Regression analyses and structural equation modeling confirmed a close association between these developments. These results inform our theoretical understanding of the development of the self‐memory system in childhood, which may contribute to the gradual offset of childhood amnesia.

**Abbreviations**
BPVS III: British Picture Vocabulary Scale III
CFI: comparative fit index
RMSEA: root mean square error of approximation
SEM: structural equation modeling
SMS: self‐memory system
TLI: Tucker–Lewis index

You have to begin to lose your memory, if only in bits and pieces, to realise that memory is what makes our lives. Life without memory is no life at all … Our memory is our coherence, our reason, our feeling, even our action. Without it we are nothing. (Buñuel, 1984, p. 17)

The importance of memory for our sense of selfhood is a keenly felt symptom of conditions which impact memory, such as dementia (Mentzou et al., 2022). However, amnesia associated with a lack of selfhood is a natural part of the lifecycle, not only as memories fade, but as they are formed. Healthy adults looking back rarely report autobiographical memories prior to the age of 2 years, a period of infantile amnesia, which gradually recedes across early to middle childhood (Henri & Henri, 1898; Pillemer & White, 1989; Tustin & Hayne, 2010). Although memories can be formed in this period (Bauer, 2015), they rarely enter our life narrative, which instead tends to prioritize memories from the heady period of adolescence and young adulthood, a bias known as the “reminiscence bump” (Rathbone et al., 2008). Thus, the rise and fall of memory for events we have experienced during our lifetime can be linked to our changing cognitive capacities, but also to our evolving sense of identity or selfhood.

Indeed, theorists have argued that until a child forms a self‐concept, as captured by the onset of mirror self‐recognition at around 2 years, autobiographical reflection on events as “something which happened to me” is a logical impossibility (Howe et al., 2003; Howe & Courage, 1993, 1997, 2004). Moreover, as reflected in Conway's (2005) “self‐memory system” (SMS), the likelihood of an event entering our life narrative as an adult is dependent partly on the extent to which it can be easily elaborated and organized into our existing semantic and autobiographical self‐knowledge. The SMS comprises our store of self‐knowledge and a goal‐sensitive “working self,” which controls access to this knowledge base. The SMS system is self‐populating in the sense that the working self executes attention and action, leading directly to the episodic event processing that populates the long‐term self (Conway et al., 2004; Conway & Pleydell‐Pearce, 2000). As a result, the human experience of self is intricately related to memory on a variety of levels. Despite the established bidirectional links between self and memory in adulthood, and the evident chronological connection between the onset of selfhood and the offset of infantile amnesia, there has been limited exploration of the *development* of the SMS (Ross et al., 2020). This is an important research gap, since growth in the self‐concept offers an intuitive explanation for the gradual offset of amnesia across early to middle childhood. The SMS described by Conway and Pleydell‐Pearce (2000), Conway et al. (2004), and Conway (2005) cannot be expected to be fully functional until sufficient semantic and autobiographical self‐knowledge has been amassed to provide an organizational structure to support episodic memory.

The mature self‐concept unifies past, current (and future, see Conway et al., 2019), real, and ideal selves. However, it is well established that this concept takes time to build, in terms of both volume of experience and complexity of self‐reflection. Classic research suggests that the information contained in a child's self‐concept undergoes qualitative shifts between early and late childhood, changing from a focus on discrete concrete features and possessions (e.g., “I have brown hair,” “I have a skateboard”) to abstract psychological characteristics derived from more global reflection on the self (e.g., “I am ambitious,” “I am friendly”; Eder, 1989, 1990; Montmayor & Eisen, 1977). Moreover, young children tend to offer unrealistically positive assessments when asked to self‐evaluate (e.g., “I am the best at everything”; Harter & Pike, 1984). As a result, the self‐concept in early childhood may be considered both fractured (consisting of a discrete collection of concrete facts about the self) and lacking in complexity (failing to differentiate between real and ideal selves). However, across middle to late childhood, the self‐concept becomes more organized and accurate, separating into differentiated domains of competence (Harter & Pike, 1984; Marsh, 1984, 1989). Although there is ongoing debate concerning how growth in the complexity of self‐knowledge might best be measured (e.g., see Cimpian et al., 2017), this age‐related change in the elaboration and organization of the self‐concept has been consistently replicated (Byrne & Shavelson, 1996; Fredricks & Eccles, 2002; Jacobs et al., 2002; Marsh & Ayotte, 2003).

Age‐related development in the capacity for binding information to autonoetic experience is also well established (Raj & Bell, 2010). In order for an event to enter the autobiographical knowledge base, episodic memory for the event must be bound with contextual information concerning subjective experience, and an ability to reflect on the source of this experience as one's own (Bauer, 2015; Hayne, 2004; Johnson et al., 1993; Newcombe et al., 2000; Perner & Ruffman, 1995; Raj & Bell, 2010; Welch‐Ross, 1995). Growth in episodic memory across early to late childhood is reflected by age‐related increases in memory specificity (Nuttall et al., 2014), binding (Sluzenski et al., 2006), source monitoring ability (Chalmers, 2014; Riggins, 2014), and the volume of information provided in event narratives (Hayne et al., 2011). Development in these areas may also be reflected in the increased volume of surviving autobiographical memories across early to middle childhood relative to infancy. Conway et al. (2005) used retrospective life narrative reports across the globe to map a lifespan retrieval curve, noting that for their adult sample, autobiographical memories increased in volume across early to late childhood, such that childhood amnesia might be considered to offset at around 10 years. The volume of available autobiographical memories then peaked in emerging adulthood, and dipped across middle age, rising to the period of recency. However, there has been little direct investigation of the impact of episodic memory developments on self‐processing, particularly in childhood, where the co‐development of self and memory processes is critical to the onset of a mature SMS.

An exception to this pattern is provided by research investigating the development of self‐reference effects in memory, the tendency for humans to better recall information that has been processed in reference to the self (e.g., “does this word describe you?”), relative to other social (e.g., “does this word describe Nelson Mandela?”), or non‐social factors (e.g., “is this word in upper or lower case?”; see Rogers et al., 1977; Symons & Johnson, 1997). As reflected in SMS theory (Conway & Pleydell‐Pearce, 2000; Conway et al. 2004; Conway, 2005), this memory advantage is thought to arise from the superior depth of processing that the mature self‐concept provides as an organizational and elaborative anchor for incoming information, and from our natural tendency to attend closely to information associated with self (Cunningham & Turk, 2017; Turk et al., 2008). The self‐reference effect emerges with the onset of mirror self‐recognition at around 2 years (Grosse Wiesmann et al., 2024) and can be observed across childhood (Andrews et al., 2020; Bennett & Sani, 2008; Cunningham et al., 2013, 2014; Halpin et al., 1984; Hutchison et al., 2021; Maire et al., 2020; Pullyblank et al., 1985; Ray et al., 2009; Ross et al., 2011, 2020; Sui & Zhu, 2005), with some studies suggesting a developmental increase beyond around 8 years of age (see Hutchison et al., 2021). Applying the self‐reference paradigm to explore the early development of the SMS, Ross et al. (2020) demonstrated that 3‐ to 6‐year‐olds' source memory for self‐referenced items predicts the volume of specific detail in their autobiographical reports of early life events, which in turn predicts the volume of their semantic self‐knowledge. However, the role of the SMS in driving the significant quantitative and qualitative change in children's self‐processing and autobiographical memory observed across early to late childhood has yet to be documented.

Although typical self‐referencing tasks offer tightly controlled laboratory‐based observations of episodic memory development, they arguably lack ecological validity concerning how the SMS functions in a real‐world context. Recognizing this, Ross et al. (2020) supplemented their self‐reference paradigm with a naturalistic self‐referential encoding task that elicited an “enactment effect” (introduced by Cohen, 1981; for review see Engelkamp, 1998). Enactment paradigms test memory for actions performed by self versus another person, assessing children's capacity to episodically process their role in a real‐life event, and to bind this information with the self‐concept (i.e., produce an accurate account of “something which happened to me”; see Badinlou et al., 2017; Ross et al., 2011, 2020). The enhanced memory for self‐performed actions (i.e., the “enactment effect”) is dependent on the extent to which a child's active experience of an event aids memorability, offering a close parallel to the autonoetic processing which underpins episodic processing in everyday life. Due to large variance in the motivations and methodologies of studies employed to observe the enactment effect, its developmental trajectory is not currently clear (Hainselin et al., 2017). However, when comparing self‐ with other‐performed action memory, Ross et al. (2011, 2020) found that the enactment effect was age‐invariant across 3 to 6 years, whereas Badinlou et al. (2017) found an increase across 8 to 14 years. Notably, this developmental pattern closely parallels the trajectory of self‐ and episodic processing outlined above.

## The current study

Conway and Pleydell‐Pearce's (2000), Conway et al.'s (2004), and Conway's (2005) SMS offers a theoretical framework that potentially unites developments in self‐concept, autobiographical memory, and episodic processing across early to middle childhood. The main objective of the current study was to deliver a developmental test of SMS theory, in order to provide a unified explanation for age‐related growth in self‐knowledge and autobiographical knowledge across early to late childhood. We assessed the volume and complexity of self‐concept by asking children to provide self‐descriptions and coding the volume of concrete and abstract self‐knowledge provided (Montmayor & Eisen, 1977; Wang, 2004). The volume and specificity of the autobiographical knowledge base was similarly assessed by asking children to describe personally important life events (first day of school/nursery, last birthday) (Wang, 2004). Finally, we employed the enactment paradigm to provide a naturalistic measure of episodic memory in which autonoetic aspects of experience could be isolated.

Based on extant research, we hypothesized that children's self‐knowledge would increase in volume and complexity across three to 11 years, moving from a focus on concrete information in early childhood, to a focus on abstract information in later childhood (9–11 years) (Eder, 1989, 1990; Montmayor & Eisen, 1977). Across the same period, we expected to see increases in the volume and specificity of children's autobiographical memory reports of important life events (Fivush et al., 1995). In keeping with developmental increases in binding and source monitoring ability (Chalmers, 2014; Riggins, 2014; Sluzenski et al., 2006), we also expected to see age‐related increases in children's ability to recall their own and other's role in recent action events, improving across early, middle, and late childhood. Since binding information to own experience is central to the enactment effect, and the self‐concept provides an anchor for incoming self‐referent information, we expected to see a corresponding developmental increase in the magnitude of the enactment effect when comparing early to later childhood (Badinlou et al., 2017; Ross et al., 2020). The enactment effect is based on the unique depth of processing arising from embodied action, and whereas it is difficult to disentangle physical from cognitive depth of processing effects underlying the enactment effect (see Ratner & Foley, 2020), a focus on source memory does allow us to determine the extent to which the action has been explicitly stored as autonoetically experienced. Thus, we use the enactment task as a way to assess the children's accurate episodic encoding of experienced and witnessed events, and to separate self‐ from other‐referent aspects of event memory.

**FIGURE 1:**
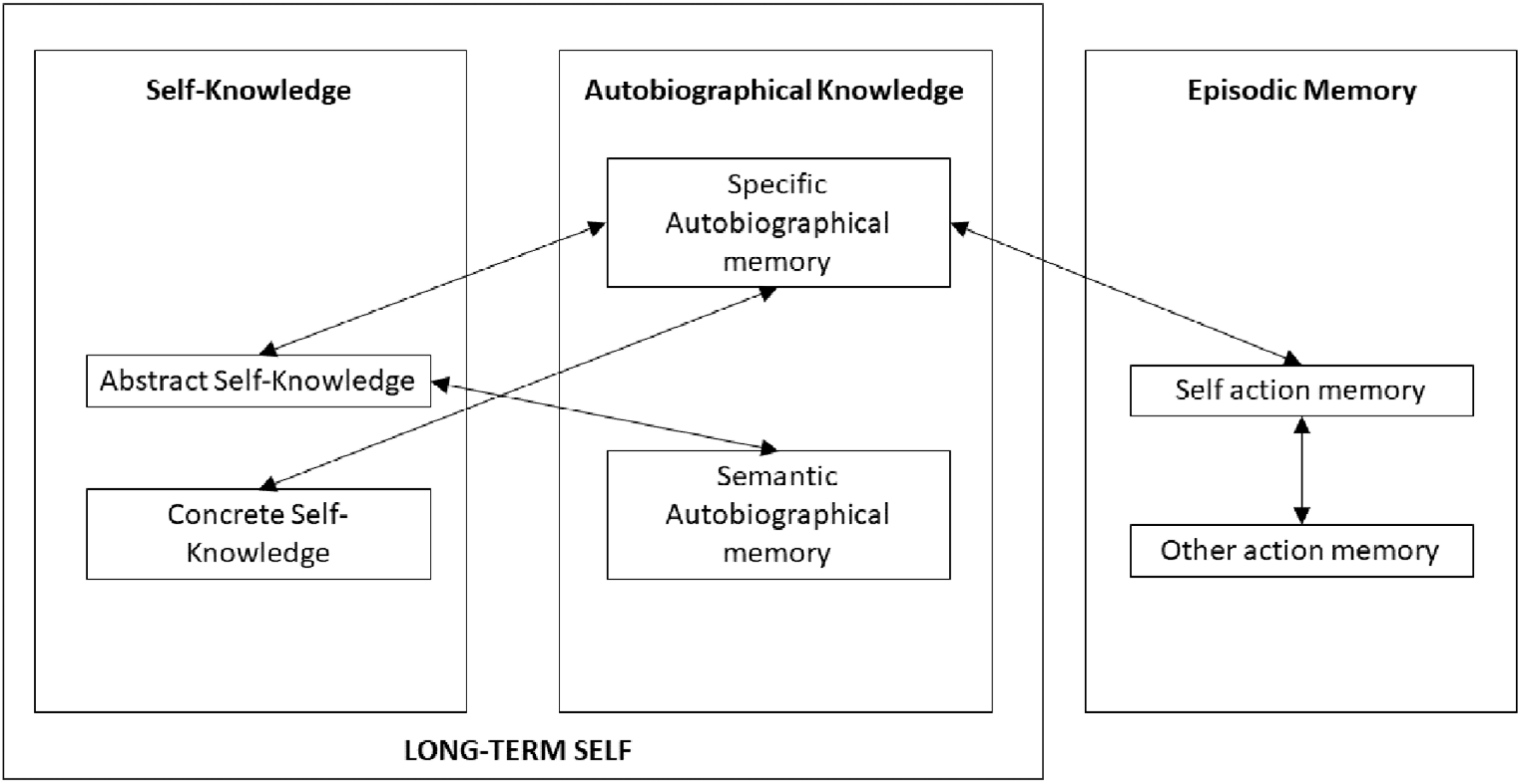
Positive predictive pathways between self and memory across 3–11 years as predicted by Conway et al.'s (2004) self‐memory system.

Having captured developmental change in self‐knowledge, autobiographical memory, and episodic processing, our ultimate aim was to test whether these developments were related to one another as described in Conway and Pleydell‐Pearce's (2000), Conway et al.'s (2004), and Conway's (2005) SMS. According to the SMS theory, to populate the self‐concept, it is necessary to gather autonoetic episodic information, and to allow for superior retrieval of this information, it is necessary to have the support of an organized and elaborated self‐concept. Thus, SMS theory suggests that self‐specific episodic processing allows for population of the autobiographical memory base, leading to an increase in semantic self‐knowledge, and strengthening the self‐concept as an anchor for incoming autobiographical information (see Figure 1). Importantly, although we cannot easily experimentally manipulate the volume and complexity of self‐knowledge to test this theory, the developmental trajectory of these capacities offers an ecologically valid testing ground to model variance in these capacities and the association between them. Thus, our data have the potential to inform our understanding of the developmental trajectory of self‐knowledge and autobiographical processing, but also to test the central assumptions of SMS theory, which has relevance across the lifespan. According to SMS theory, we should find close relationships between self‐knowledge and autobiographical knowledge comprising our “long‐term self,” a construct populated by, and supportive of, the autonoetic retention of episodic events.

## METHOD

### Participants

A sample of 379 three‐ to eleven‐year‐olds took part (54% female), comprising 122 three‐ to five‐year‐olds, *M* = 52.99, SD = 9.49; 123 six‐ to eight‐year‐olds, *M* = 90.05, SD = 9.95; and 133 nine‐ to eleven‐year‐olds, *M* = 124.84, SD = 10.02. All children were pupils at local nurseries or schools. Race and socioeconomic status data were not collected, but the children were recruited from predominantly White, lower to middle‐class Scottish areas in 2018. The children were tested individually in their place of education with the written consent of a parent or guardian, and their own assent, and the research was approved by the Universities of Dundee and Abertay Research Ethics Committees. G* Power analyses indicated that a sample of *N* = 120 per age group would achieve over 80% power to detect medium effects in ANOVA and regression analyses (including six predictors).

### Procedure

The children were tested individually in their place of education over three sessions, each separated by 1 week. They completed the enactment task and vocabulary test in session one, tasks not included in the current study in session two, and autobiographical and self‐description tasks in session three. Order effects were not anticipated given the disparate tasks used, but the order of tasks was chosen based on piloting to allow the child to gradually familiarize themselves with the researcher, at first via highly structured games, and later in free conversation. At the end of each session, the child was thanked, given a sticker, and taken back to class.

#### Enactment task

**FIGURE 2:**
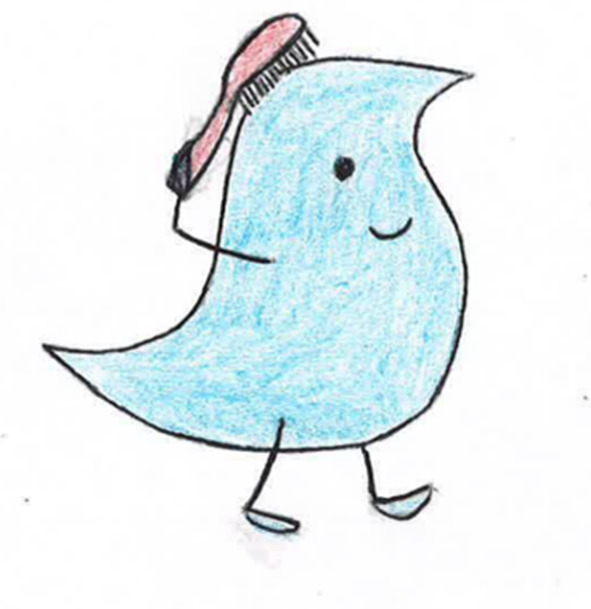
Example stimuli for the enactment task. Action list for enactment task (participant starts or experimenter starts, order counterbalanced); 1. Dance, 2. Put on a tie, 3. Wave, 4. Smell a flower, 5. Touch toes, 6. Go fishing, 7. Hammer a nail, 8. Brush hair, 9. Brush teeth, 10. Close eyes, 11. Star jump, 12. Hands on head, 13. Lick an ice cream, 14. Hands over mouth, 15. Play guitar, 16. Thumbs up, 17. Rub tummy, 18. Kick a football, 19. Fingers in ears, 20. Wag finger “no,” 21. Put on sunglasses, 22. Fly a kite, 23. Blow out candles, 24. Point.

The children completed an enactment task adapted from Ross et al. (2011). Participants were introduced to a fictional character called a “wug” (based on Gleason/Berko's (1958) illustration of a novel, age‐ and gender‐neutral character). Across a series of 24 picture cards that were revealed in turn, the wug was depicted performing everyday actions counterbalanced for object‐use and self‐directedness, see Figure 2 for action list and example stimuli.

The experimenter and participant acted out alternate actions modeled by the wug, imagining any objects depicted (e.g., performing the action of putting on a tie without a real tie being present). The order of the actions was counterbalanced across experimenter and participant. After a delay in which participants completed a vocabulary task (detailed below), they were asked to freely recall the actions, and identify the source of each (i.e., indicate whether they were performed by self or the experimenter). To provide a self‐source score, the proportion of hits for self‐actions was calculated (i.e., number of self‐items correctly attributed to being conducted by self/12 = total number of self‐actions). The equivalent score was calculated for other‐source. To correct for guessing, the proportion of false alarms was calculated for self and other actions (number of actions incorrectly recalled as being performed by self or other/12) and subtracted from raw scores to give a final, corrected score for self‐source (proportion self‐hits—proportion self‐false alarms) and other‐source (proportion other hits—proportion other false alarms). This correction method ensures that we focus on the volume of accurate source and item memory (i.e., data that fulfill the criteria for episodic recall).

#### Vocabulary task

The NIH Toolbox Vocabulary Task (see Akshoomoff et al., 2014) was used as the vocabulary task for the majority of participants (*n* = 317 three‐ to eleven‐year‐olds). This task was performed on a touchscreen laptop provided by the experimenter. Within each trial, a set of four pictures was presented onscreen simultaneously with a spoken word that described one of the pictures. Participants were then asked to select the picture that best matched the spoken word by touching the appropriate picture on the laptop screen. Due to the lack of reading or literacy requirement, this task is deemed suitable for children of all ages and abilities. Items were administered to match each participant's ability with item difficulty: the difficulty of each successive item presented is based on the current estimate of the participant's ability level, as estimated by their responses to the previously administered items on the test. Each participant was exposed to 25 trials and the task lasted on average approximately 5 min. In an unplanned task change, the remaining participants (*n* = 61 six‐ to eleven‐year‐olds) completed the British Picture Vocabulary Scale III (BPVS III) (Dunn et al., 2009), due to discontinuation of support for the NIH Toolbox measure. The BPVS III is essentially a manual equivalent of the NIH Toolbox Vocabulary Task, involving the experimenter saying a word and the child identifying its meaning by selecting a picture from four picture options. The time taken to complete the BPVS is more variable (10–15 min), since the child continues until a key number of errors are made. Note, this increased delay may have made recall in the enactment task more challenging for the subsample of *N* = 61 six‐ to eleven‐year‐old children whose vocabulary was assessed using the BPVS III. However, when this subgroup was removed from the sample the main effect of enactment, *F*(1, 314) = 63.72, *p* < .001, $\eta_{p}^{2}$ = .17, and interaction between age and enactment, *F*(2, 314) = 7.50, *p* < .001, $\eta_{p}^{2}$ = .05, remained unchanged. Moreover, the enactment effect remained significant for the smaller subsample, *F*(1, 60) = 9.99, *p* = .002, $\eta_{p}^{2}$ = .14. Both measures assess receptive vocabulary and have a standardized scoring system resulting in each child receiving an age‐corrected score with a normative mean of 100 and a standard deviation of 15; thus, the vocabulary scores were simply combined into one variable. As a result of this disparity in testing instruments, which was confounded with age, correlational analyses including this variable should be interpreted with caution.

#### Autobiographical memory and self‐descriptions

To elicit retrospective autobiographical event narratives, the experimenter asked the children, “What can you remember about your first day of school/nursery?” and “What can you remember about your last birthday?” These events were chosen because they are considered memorable from a cultural perspective, and they objectively happened to each child in the recent past. Following each response, the experimenter used standard prompts such as “What else can you remember?” and “Is there anything else?” until the child indicated by speech or gesture that they had finished their narrative.

Propositions, described by Fivush et al. (1995; see also Wang, 2004) as subject‐verb propositions, were used as the coding unit, with each new proposition counting as a new unit (e.g., “I dance” was one unit; “I dance and swim” was two units). The propositions that made up children's memory reports were coded as either *specific* if they referred to a memory that occurred at a particular point in time (e.g., I had a Spiderman birthday cake) or *semantic* for memories that were likely script based, referring to events that took place on multiple occasions or happened regularly (e.g., I had a birthday cake; Wang, 2004). A count of each response type was calculated to provide a meaningful measure of both volume and specificity of autobiographical reports.

An open‐ended question was used to elicit the child's self‐description. The researcher asked the child “I wonder if you can tell me some things all about you, some things that would describe [child's name] to me?” Prompts such as “What else could I write about you?” were used by the researcher until the child indicated by speech or gesture that they had finished. Interviews were transcribed verbatim for coding post‐interview (Wang, 2004). Responses referring to qualities, opinions or traits were coded as abstract (e.g., “I love to jump”); and responses referring to physical traits or facts (e.g., “I have brown eyes”; “I have a sister”) were coded as concrete (Wang, 2004). A count of each response type was calculated to provide a meaningful measure of both volume and quality of self‐knowledge.

The autobiographical memory and self‐description tasks were completed in this fixed order to replicate Wang (2004), and since our piloting of the tasks indicated that the autobiographical memory task was a more effective conversation opener than the free self‐description task, perhaps as the task to recall an event is more common and concrete to children than being asked to reflect more broadly on themselves. Inter‐rater reliability for coding within the autobiographical memory task and self‐description task was established by having the data coded by two independent raters (authors 1 and 2) and yielded a robust Cohen's kappa score (*k* = .97 overall, *k* = .95 specific vs. semantic, *k* = .99, concrete vs. abstract).

#### Data analysis

To characterize the development of enactment effects, autobiographical memory, and self‐knowledge, we ran a series of repeated‐measures ANOVAs in SPSS V28 comparing corrected action memory for self versus corrected action memory for other; volume of specific memory details versus volume of semantic memory details; and volume of abstract self‐descriptions versus volume of concrete self‐descriptions. Note, specific autobiographical memory, total self‐description scores, and enactment task source scores of the 3‐ to 6‐year‐olds in the current study (*n* = 186) were also included in analyses reported by Ross et al. (2020). However, the breakdown between concrete and abstract self‐descriptions and semantic versus specific autobiographical memory has not previously been analyzed or published, nor has any autobiographical memory data from the remainder of the current sample (i.e., 193 seven‐ to eleven‐year‐olds). Our analyses are confirmatory, based on predicted developmental patterns.

Age group was included in all ANOVAs as a between subject's factor representing early (3–5 years) middle (6–8 years) and later childhood (9–11 years). Our age groups were theoretically driven to reflect different stages in self‐development: early, middle, and late childhood. The focus of our first set of analyses was to replicate the age‐related switch from concrete to abstract self‐description and to determine whether this was accompanied by qualitative age‐related change in other aspects of the SMS—the emergence of memory specificity and enactment effects. Where interactions were significant, these were broken down via simple effects analyses, and difference scores were calculated to allow clear observation of the magnitude of effects via planned Bonferroni comparisons. Due to the unplanned task change whereby children completed only one of two different vocabulary measures, vocabulary score was not included in this analysis. However, for completeness, we did run vocabulary score as a covariate to check that controlling for this factor did not affect any of our reported patterns, finding that it did not.

To characterize how developmental changes in task performance were related, we calculated zero‐order correlations between all variables, followed by a series of regression analyses separately predicting performance on the enactment task (self‐action memory, other‐action memory), autobiographical memory task (specific memory, semantic memory) and self‐description task (abstract self‐description, concrete self‐description). In each regression, we included the remaining five outcome measures and age in months as potential predictors. These analyses allowed us to determine whether, controlling for age and general source monitoring ability (as captured in source memory for other), there are self‐specific predictive relationships between self‐source monitoring, autobiographical memory, and self‐description as illustrated in Figure 1.

Finally, we used structural equation modeling (SEM) in SPSS AMOS V28 to determine the extent to which Conway and Pleydell‐Pearce's (2000), Conway et al.'s (2004), and Conway's (2005) SMS offers a good fit for our data when controlling for measurement error and the common association of age. SEM enabled associations between multiple outcomes to be assessed simultaneously rather than relying on separate regressions for each relationship (Kline, 2005). Absolute fit indices were employed to investigate the model fit (McDonald & Ho, 2002), including chi‐square (with a non‐significant outcome representing a good model fit), root mean square error of approximation (RMSEA; with a value of < or =.05 indicating good model fit), and the comparative fit index (CFI) and Tucker–Lewis index (TLI) (both with a value greater than .9 representing good fit).

These analysis techniques involve the assumption of normality. Although there was evidence of moderate non‐normality in the data (as is common in developmental samples), linear models are thought to be robust to this (e.g., see Blanca Mena et al., 2023), particularly where the sample is well powered, and conservative Greenhouse–Geisser tests are reported. To account for non‐normality within our structural equation analysis, maximum‐likelihood estimates were used alongside 5000 bootstrap samples and the Bollen–Stine bootstrap test to confirm that the model fit was robust to non‐normality.

## RESULTS

### Developmental patterns in enactment, autobiographical memory, and self‐descriptions

#### Enactment

Table 1 shows raw recall and source recall in the enactment task, with paired sampled *t*‐tests confirming an enactment effect for both item recall and source recall.

**TABLE 1:** Average item recall and source memory in the enactment task (uncorrected).

	*M* (SD)	Paired *t t*‐test
Recall for self‐actions	2.71 (1.71)	*t*(377) = 7.97, *p* < .001
Recall for other actions	2.00 (1.42)	
Source memory for self‐actions	2.59 (1.71)	*t*(377) = 8.83, *p* < .001
Source memory for other actions	1.83 (1.46)	

**FIGURE 3:**
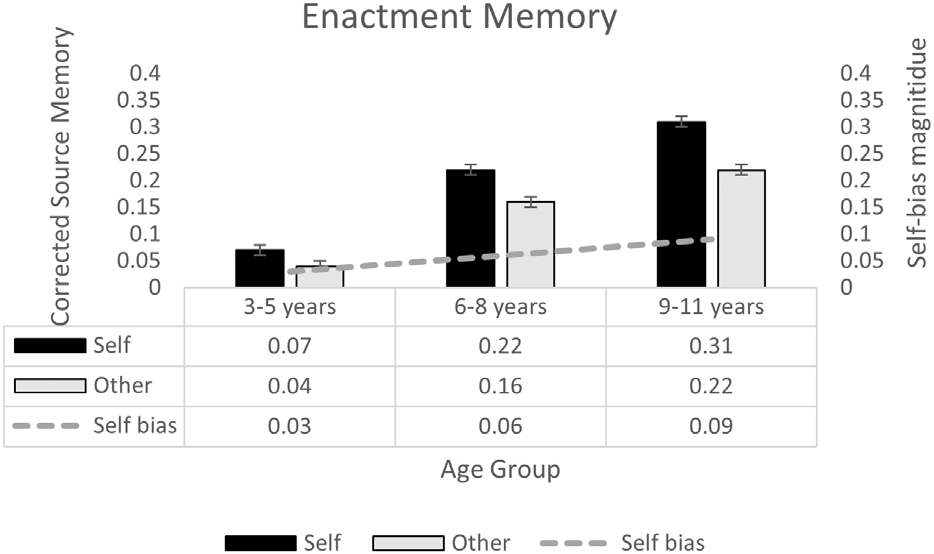
Performance in the enactment task, split by encoding condition and age group. Bars show corrected source memory (and standard error) for each condition (self, other) and age group. Line shows the magnitude of self‐bias (memory for self‐action minus memory for other‐action).

Figure 3 shows children's performance in the enactment task based on corrected source memory, split by encoding condition and age group. A 2 (Referent: Self, Other) × 3 (Age Group: 3–5 years, 6–8 years, 9–11 years) repeated‐measures ANOVA indicated a main effect of encoding condition in the enactment task, *F*(1, 375) = 64.82, *p* < .001, $\eta_{p}^{2}$ = .15, reflecting children's tendency to better recall actions that they had performed, *M* = 0.20, SE = .01, relative to actions they had witnessed the researcher perform, *M* = 0.14, SE = .01. Children's action memory significantly improved with age, *F*(2, 375) = 187.24, *p* < .001, $\eta_{p}^{2}$ = .50, with simple effects tests and postdoc Bonferroni comparisons indicating significant age‐related gains in self, *F*(2, 375) = 152.11, *p* < .001, $\eta_{p}^{2}$ = .45, and other‐action memory, *F*(2, 375) = 79.69, *p* < .001, $\eta_{p}^{2}$ = .30, significant at every developmental step (*p* < .001). There was a significant interaction between age and encoding condition *F*(2, 375) = 9.26, *p* < .001, $\eta_{p}^{2}$ = .05. Simple effects tests indicated that the enactment effect was significant in middle and late childhood (both *p* < .001), but fell short of significance for the youngest age group (*p* = .077). To confirm age‐related change in the magnitude of the enactment effect, we calculated a self‐bias score by subtracting memory for other‐performed actions from memory for self‐performed actions (shown in the line in Figure 3) and entered this as the dependent variable in a univariate ANOVA with age group as a between‐subjects factor. This analysis confirmed a main effect of age group, *F*(2, 375) = 9.26, *p* < .001, $\eta_{p}^{2}$ = .05, reflecting age‐related change in the magnitude of the enactment effect. This magnitude differed significantly when comparing the early (*p* < .001) and middle childhood (*p* = .041) to late childhood, but not when comparing early and middle childhood (*p* = .233).

#### Autobiographical memory

**FIGURE 4:**
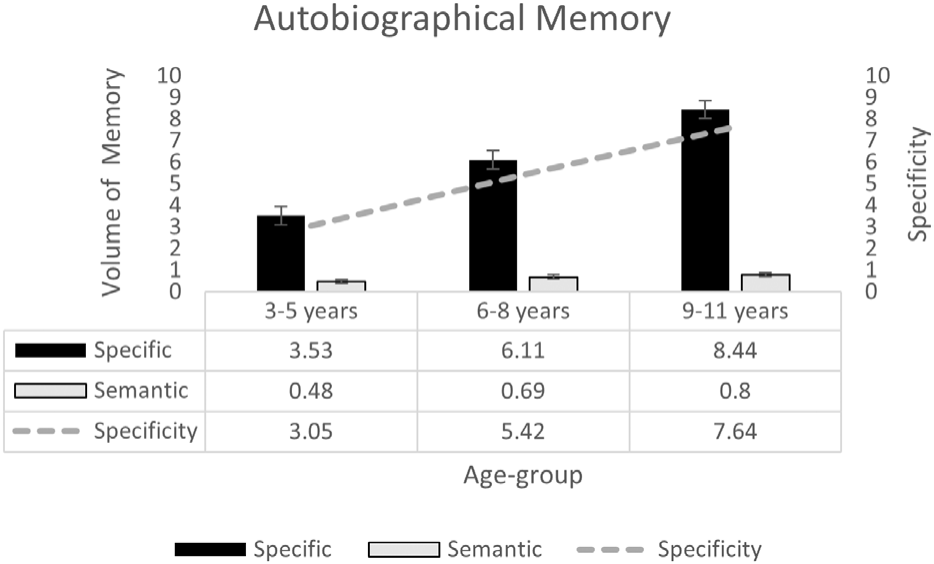
Performance in autobiographical memory task, split by memory type, and age group. Bars show the volume of memory (and standard error) in the autobiographical memory task for each memory type (specific, semantic) and age group. Line shows specificity (volume of specific memory minus volume of semantic memory).

Figure 4 shows the volume of specific and semantic memory reports children offered, split by age group. A 2 (Memory type: specific, semantic) × 3 (Age Group: 3–5 years, 6–8 years, 9–11 years) repeated‐measures ANOVA indicated a main effect of memory type, *F*(1, 375) = 482.13, *p* < .001, $\eta_{p}^{2}$ = .56, reflecting children's tendency to offer more specific, *M* = 6.03, SE = .24, than semantic, *M* = 0.660, SE = .05, memories. The volume of information given significantly increased with age, *F*(2, 375) = 34.77, *p* < .001, $\eta_{p}^{2}$ = .16, with simple effects tests indicating significant age‐related gains in specific, *F*(2, 375) = 33.68, *p* < .001, $\eta_{p}^{2}$ = .15, and semantic memory, *F*(2, 375) = 3.17, *p* = .043, $\eta_{p}^{2}$ = .02. For specific memory, Bonferroni comparisons indicated significant increases at each developmental step (all *p* < .001); however, for semantic memory, the increase was only apparent when comparing early to late childhood (*p* = .039), and not when comparing early to middle (*p* = .344) or middle to late childhood (*p* = 1.00). There was a significant interaction between age and memory type *F*(2, 375) = 29.62, *p* < .001, $\eta_{p}^{2}$ = .14. Simple effects tests indicated that the bias toward specific memories was consistent for all age groups (all *p* < .001). However, to explore age‐related increases in specificity, we calculated a specificity score by subtracting the volume of semantic memories offered from the volume of specific memories (shown in the line in Figure 4), and entered this as the dependent variable in a univariate ANOVA with age group as a between‐subjects factor. This analysis confirmed a main effect of age group, *F*(2, 375) = 29.62, *p* < .001, $\eta_{p}^{2}$ = .14, reflecting a steady age‐related increase in autobiographical memory specificity, significant with each developmental step (all *p* < .001).

#### Self‐description

**FIGURE 5:**
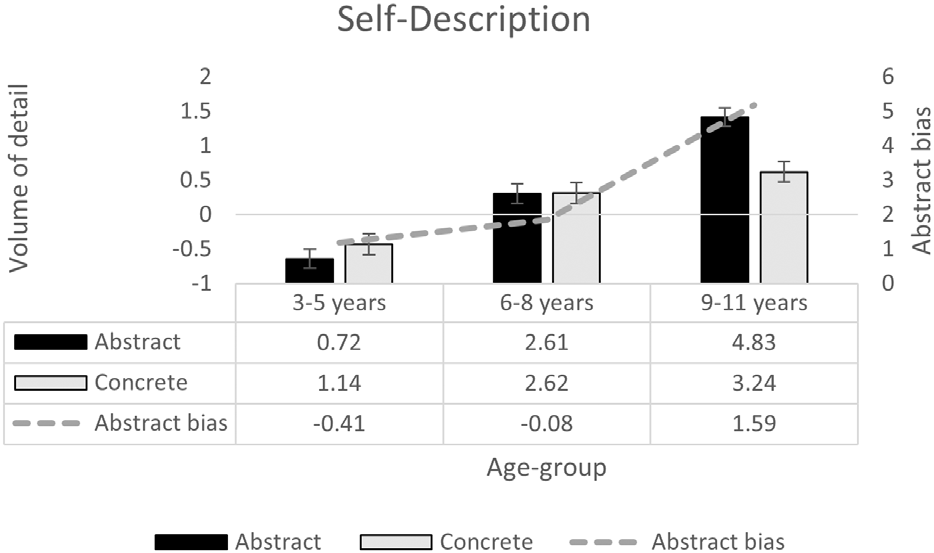
Performance in self‐description task, split by description type, and age group. Bars show the volume of detail (and standard error) in the self‐description task for each description type (abstract, concrete) and age group. Line shows bias toward abstract over concrete details.

Figure 5 shows the volume of abstract and concrete self‐descriptions children offered, split by age group. A 2 (Self‐description type: abstract, concrete) × 3 (Age Group: 3–5 years, 6–8 years, 9–11 years) repeated‐measures ANOVA indicated no main effect of self‐description type, *F*(1, 375) = 3.34, *p* = .068, $\eta_{p}^{2}$ = .01 (abstract: *M* = 2.72, SE = .16; concrete: *M* = 2.33, SE = .17). However, the volume of information given significantly increased with age, *F*(2, 375) = 47.41, *p* < .001, $\eta_{p}^{2}$ = .20, with simple effects tests indicating significant age‐related gains in both abstract, *F*(2, 375) = 54.70, *p* < .001, $\eta_{p}^{2}$ = .23, and concrete self‐descriptions, *F*(2, 375) = 12.71, *p* < .001, $\eta_{p}^{2}$ = .06. For abstract self‐descriptions, Bonferroni comparisons indicated significant increases at each developmental step (*p* < .001). However, for concrete self‐descriptions, the increase was weaker and only apparent when comparing the early to middle (*p* = .002) and late childhood (*p* < .001), and not when comparing middle and late childhood (*p* = .430). There was a significant interaction between age and self‐description type *F*(2, 375) = 8.56, *p* < .001, $\eta_{p}^{2}$ = .04. Simple effects tests confirmed that whereas the younger age groups gave an equivalent volume of abstract and concrete self‐descriptions (3–5 years, *F*(1, 375) = 1.26, *p* < .262, $\eta_{p}^{2}$ = .003; 6–8 years, *F*(1, 375) = 0.000, *p* = .98, $\eta_{p}^{2}$ < .000), the eldest children showed a bias toward abstract over concrete self‐description, *F*(1, 375) = 19.81, *p* < .001, $\eta_{p}^{2}$ = .05.

To explore age‐related increases in the bias toward abstract information, we subtracted the volume of concrete self‐descriptions from abstract self‐descriptions (shown in the line in Figure 5) and entered this as the dependent variable in a univariate ANOVA with age group as a between‐subjects factor. This analysis confirmed a main effect of age group, *F*(2, 375) = 8.56, *p* < .001, $\eta_{p}^{2}$ = .04. Planned comparisons confirmed the pattern was unchanged between early and middle childhood (*p* = 1.00), but differed by late childhood, when abstract self‐descriptions were dominant (early: *p* < .001, middle: *p* = .006).

#### Relations between enactment, autobiographical memory, and self‐description

Table 2 shows zero‐order Pearson's correlations between the two enactment scores (self, other), two autobiographical memory scores (specific, semantic), and two self‐description scores (abstract, concrete), along with age in months. For completeness, standardized receptive vocabulary scores were included, although these need to be interpreted with caution as these data comprise scores from two different tasks. Confirming age‐related growth in self and memory, all variables show a positive relationship with age. Vocabulary related positively to specific autobiographical memory. Memory for self‐ and other‐performed action was related to specific but not semantic autobiographical memory, as might be expected by the specificity required of the enactment task, and the relatively low variance in the semantic data. Memory for both action types was also related to the volume of concrete and abstract self‐descriptions provided. Finally, both semantic and specific autobiographical memory were related to abstract and concrete self‐knowledge.

**TABLE 2:** Summary of correlations for enactment, autobiographical memory, and self‐knowledge, including relations to age and receptive vocabulary.

	Self action memory	Other‐action memory	Specific autobiographical memory	Semantic autobiographical memory	Abstract self‐description	Concrete self‐description	Age	Vocab
Self‐action memory	—	.495**	.387**	.049	.384**	.244**	.683**	.051
Other‐action memory		—	.315**	.086	.284**	.186**	.562**	.019
Specific autobiographical memory			—	.163**	.527**	.404**	.408**	.133*
Semantic autobiographical memory				—	.257**	.131*	.161*	−.016
Abstract self‐description					—	.294**	.501**	.097
Concrete self‐description						—	.246**	.074
Age							—	−.046

* *p* < .05;

** *p* < .001.

Additional analyses based on the difference scores computed above indicated that the magnitude of the enactment effect *r* = .21, *p* < .001, and autobiographical memory specificity, *r* = .38, *p* < .001, increased with age, and related to one another, *r* = .13, *p* = .011, and to abstract self‐knowledge (*r* = .15, *p* = .004, *r* = .48, *p* < .001, respectively). Autobiographical memory specificity, *r* = .38, *p* < .001, but not enactment effect magnitude, *r* = .09, *p* = .084, also related to the volume of concrete self‐knowledge.

To determine the independent relationships between our key variables controlling for the common association of age, we ran a series of regression analyses predicting the outcome of enactment, autobiographical memory, and self‐description tasks. In each regression, we included the remaining five outcome measures and age as potential predictors. The results of these analyses are summarized in Table 3. Variance inflation factor levels ranged between 1.055 and 2.169, indicating multicollinearity was not an issue. To focus on our main hypotheses, preserve power, and avoid the potential confound of children completing one of two vocabulary tests, vocabulary was not included in the reported regression analyses. However, the regression patterns were not substantially changed by including vocabulary, and this factor did not emerge as a significant predictor in any analysis.

**TABLE 3:** Multiple linear regression analyses predicting task performance.

	*B*	*t*‐test
**Predicting self‐action memory**		
*F*(6, 377) = 62.506, *p* < .001, *r* ^2^ = .503		
Age	0.003	*t* = 11.142, *p* < .001**
Other‐action memory	0.171	*t* = 3.294, *p* = .001**
Specific autobiographical memory	0.003	*t* = 2.167, *p* = .031*
Semantic autobiographical memory	−0.012	*t* = −2.113, *p* = .035*
Abstract self‐description	0.001	*t* = 0.447, *p* = .655
Concrete self‐description	0.002	*t* = 1.121, *p* = .263
**Predicting other‐action memory**		
*F*(6, 377) = 32.670, *p* < .001, *r* ^2^ = .346		
Age	0.002	*t* = 6.695, *p* < .001**
Self‐action memory	0.166	*t* = 3.294, *p* = .001**
Specific autobiographical memory	0.002	*t* = 1.733, *p* = .084
Semantic autobiographical memory	0.001	*t* = 0.136, *p* = .892
Abstract self‐description	−0.002	*t* = −0.973, *p* = .331
Concrete self‐description	0.001	*t* = 0.318, *p* = .331
**Predicting specific autobiographical memory**		
*F*(6, 377) = 37.815, *p* < .001, *r* ^2^ = .379		
Age	0.005	*t* = 0.450, *p* = .653
Self‐action memory	4.238	*t* = 2.167, *p* = .031*
Other‐action memory	3.439	*t* = 1.733, *p* = .084
Semantic autobiographical memory	0.098	*t* = 0.460, *p* = .646
Abstract self‐description	0.528	*t* = 7.382, *p* < .001**
Concrete self‐description	0.354	*t* = 5.532, *p* < .001**
**Predicting semantic autobiographical memory**		
*F*(6, 377) = 5.498, *p* < .001, *r* ^2^ = .082		
Age	0.004	*t* = 1.584, *p* = .114
Self‐action memory	−1.006	*t* = −2.113, *p* = .035*
Other‐action memory	0.066	*t* = 0.136, *p* = .892
Specific autobiographical memory	0.006	*t* = 0.460, *p* = .646
Abstract self‐description	0.063	*t* = 3.435, *p* < .001**
Concrete self‐description	0.018	*t* = 1.092, *p* = .276
**Predicting abstract self‐description**		
*F*(6, 377) = 41.233, *p* < .001, *r* ^2^ = .400		
Age	0.038	*t* = 5.491, *p* < .001**
Self‐action memory	0.596	*t* = 0.447, *p* = .655
Other‐action memory	−1.312	*t* = −0.973, *p* = .331
Specific autobiographical memory	0.243	*t* = 7.382, *p* < .001**
Semantic autobiographical memory	0.489	*t* = 3.435, *p* < .001**
Concrete self‐description	0.056	*t* = 1.236, *p* = .217
**Predicting concrete self‐description**		
*F*(6, 377) = 13.680, *p* < .001, *r* ^2^ = .181		
Age	0.001	*t* = 0.133, *p* = .894
Self‐action memory	1.715	*t* = 1.121, *p* = .263
Other‐action memory	0.493	*t* = 0.318, *p* = .751
Specific autobiographical memory	0.215	*t* = 5.532, *p* < .001**
Semantic autobiographical memory	0.181	*t* = 1.092, *p* = .276
Abstract self‐description	0.074	*t* = 1.236, *p* = .217

* *p* < .05;

** *p* < .001.

The regression model predicting memory for self‐performed actions from other‐action memory, autobiographical memory, self‐descriptions, and age accounted for 50% of the variance, with independently significant contributions from other‐performed action and both semantic and specific autobiographical memory. Strong memory for other‐performed actions and specific event details predicted strong memory for self‐performed actions, whereas semantic autobiographical memory made a negative contribution to this model. Specific and semantic autobiographical details had a positive zero‐order correlation. Nonetheless, when entered together as predictors in our regression, the additional predictive variance captured by semantic memories may reflect the fact that these memories by definition contained less specific detail, and so consequently might be expected to relate to poorer source memory. A parallel model runs to predict memory for other‐performed actions from self‐action memory, autobiographical memory, self‐descriptions, and age accounted for 35% of the variance, with self‐performed action memory emerging as the only independently significant predictor.

The regression model predicting specific autobiographical memory from semantic autobiographical memory, action memory, self‐descriptions, and age accounted for 38% of the variance, with self‐performed action memory, and abstract and concrete self‐descriptions offering independent and positive contributions to the model. This indicates that children who offered a greater volume of self‐referent information in both memory reports and self‐descriptions were likely to report more specific details of autobiographical life events. However, a parallel model runs to predict semantic autobiographical memory from specific autobiographical memory, action memory, self‐descriptions and age was less successful, accounting for a significant 8% of variance, with negative contributions from self‐source monitoring and a small positive contribution from abstract self‐description.

The regression model predicting abstract self‐knowledge from concrete self‐knowledge, action memory, autobiographical memory and age accounted for 40% of the variance, with age and specific and semantic memory offering independent contributions to the model. In this case, the contribution of semantic memory was positive, indicating that the older children were, and the greater volume of semantic and specific information they gave in their event reports, the more likely they were to provide abstract self‐descriptions. The parallel model runs to predict concrete self‐knowledge from abstract self‐knowledge, action memory, autobiographical memory, and age accounted for only 18% of the variance, with a positive contribution only from specific autobiographical memory.

The positive independent predictive pathways suggested by these regression analyses closely match the predictions of the SMS shown in Figure 1, where episodic memory for events (as captured by our enactment task) is closely associated with autobiographical memory, which in turn is closely associated with self‐knowledge.

#### Modeling the SMS

**FIGURE 6:**
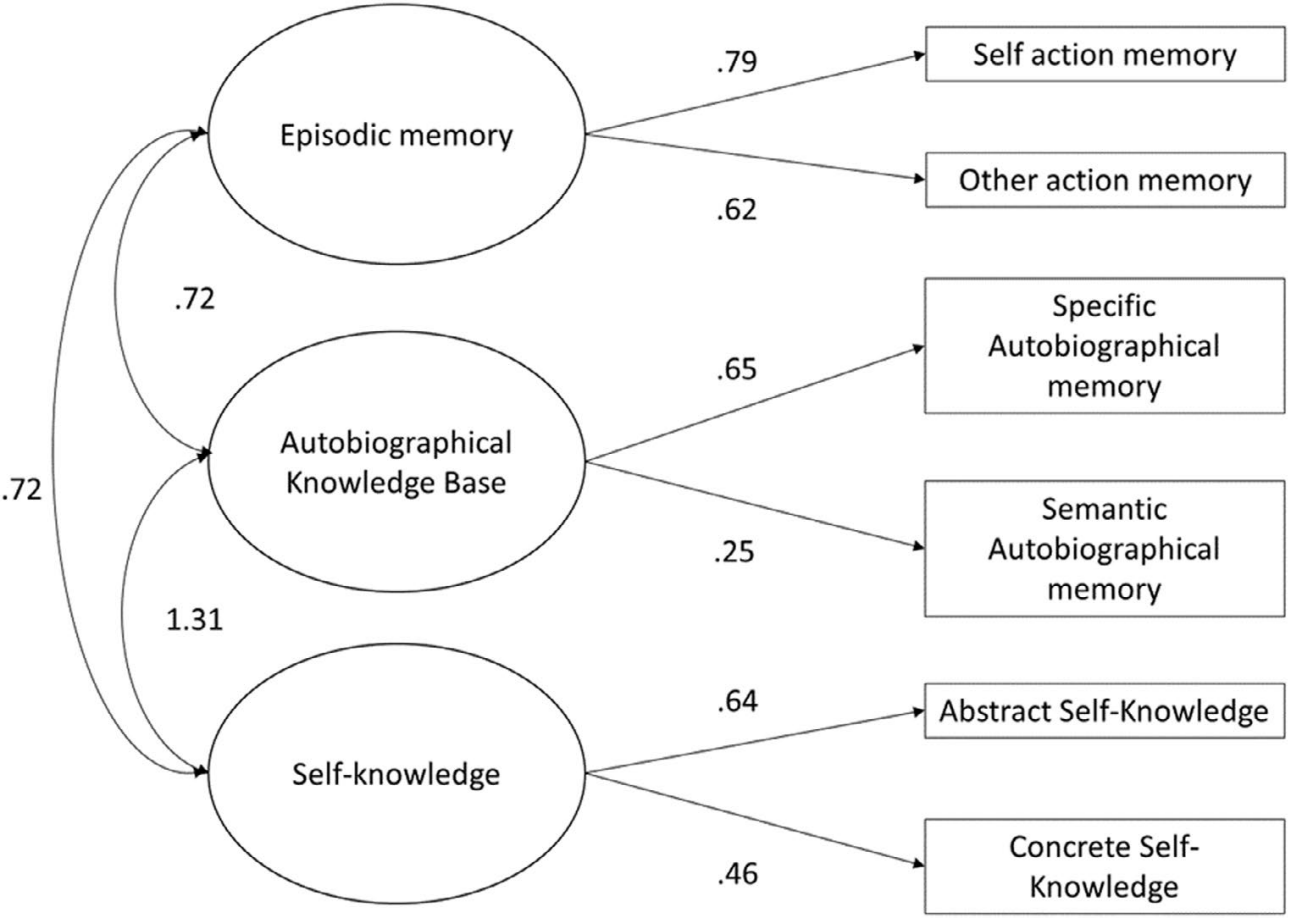
Self‐memory system model development, with self‐knowledge and autobiographical knowledge as separate latent variables. Correlations are shown for bidirectional arrows, and standardized regression weights for directional arrows. However, this model uncovered multicollinearity in self‐description and autobiographical memory resulting in failure to converge.

**FIGURE 7:**
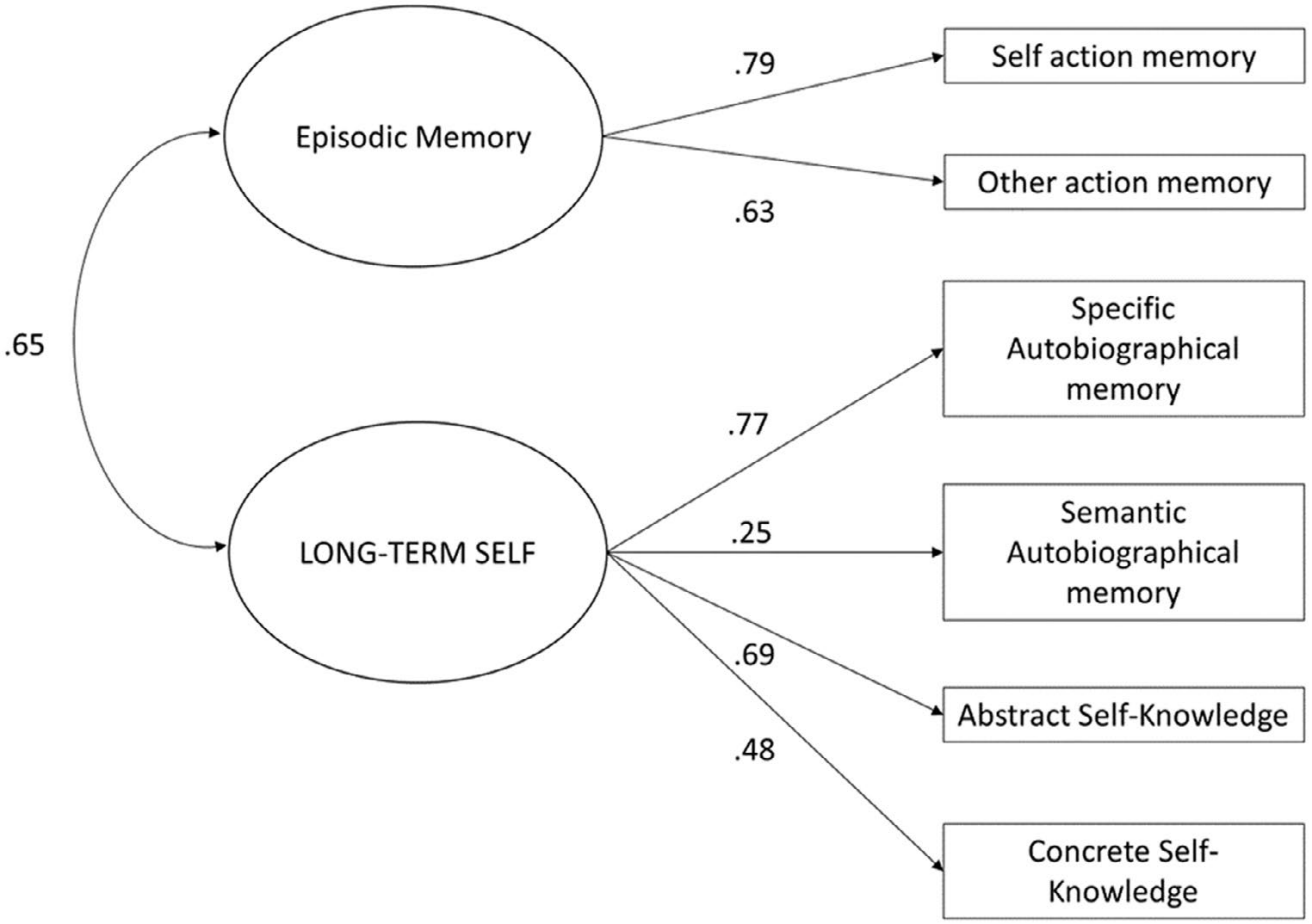
Final self‐memory system model, with self‐knowledge and autobiographical knowledge encompassed by the long‐term self as a latent variable. Correlations are shown for bidirectional arrows, and standardized regression weights for directional arrows. This final model appropriately addressed multicollinearity in self‐description and autobiographical memory and represented a good fit.

To confirm the proposed relationship between our developmental data and the SMS, we ran a structural equation model using SPSS AMOS V28. Our first model, with standardized regression coefficients, is shown in Figure 6 (error terms were fitted to all endogenous variables but are omitted to aid readability). Abstract and concrete self‐knowledge became manifest variables for self‐knowledge as a latent variable, and specific and semantic autobiographical memory became the manifest variables representing the latent autobiographical knowledge base. Latent episodic memory was represented by self‐ and other‐action memory. We predicted bidirectional covariance between all three latent variables, all paths were significant, and the model fit indices suggested an excellent fit, *χ* ^2^ = 9.08, *p* = .169, CFI = .99, TLI = .98, RMSEA = .037. However, the covariance between the autobiographical knowledge base and self‐knowledge (>1) suggested multicollinearity between the latent variables of self‐knowledge and autobiographical knowledge, and the covariance matrix would not converge, making the fit indices questionable. To address this in a theoretically driven manner, we adopted Conway et al.'s (2004) overarching framework of the “long‐term self,” which encompasses the self and autobiographical knowledge bases, using this as the latent variable for all self‐knowledge and autobiographical knowledge variables, as shown in Figure 7. This allowed the model to converge, offering a good fit *χ* ^2^ = 14.50, *p* = .070, CFI = .98, TLI = .97, RMSEA = .046, with all pathways remaining significant, *p* < .001. Bootstrapped confidence intervals showed that the standardized regression weights were robust, and the Bollen–Stine bootstrapping test was non‐significant *p* = .259 indicating that the model fit could be considered reliable. The model shows significant covariance between episodic memory and the long‐term self, as predicted by the SMS.

Finally, to account for age as a potential confounding factor explaining the relationship between the long‐term self and episodic memory, we considered age in months as the ultimate exogenous variable, predicting the latent variables of episodic memory and the long‐term self. This allowed us to control for the common association with age when visualizing direct pathways between self and memory. Two unidirectional mediation models were run, interchanging the mediator and the outcome. Note, the model fit is identical in both scenarios since the parameters are equivalent—a reduced but acceptable, *χ* ^2^ = 28.29, *p* = .005, CFI = .98, TLI = .96, RMSEA = .060. Bootstrapped confidence intervals showed that the standardized regression weights were robust to bootstrapping, and the Bollen–Stine bootstrapping test was non‐significant *p* = .114, indicating that the model fit could be considered reliable.

**FIGURE 8:**
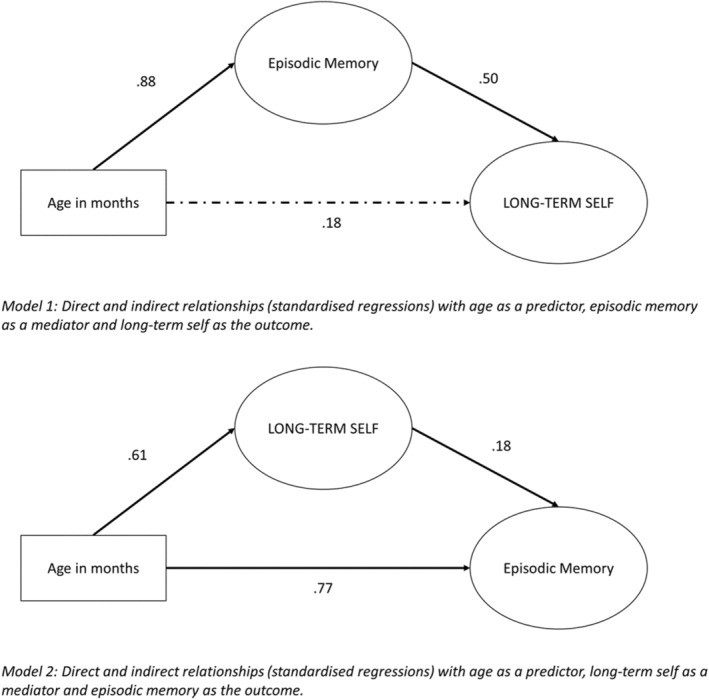
Modeling relations between the long‐term self and episodic memory with age as an exogenous predictor, alternating episodic memory and long‐term self as mediator and outcome variables. Factor loadings for manifest variables in the inner model are as shown in Figure 7. In this figure, standardized regression weights for shown for directional arrows, with significant regression pathways represented by a solid line, and non‐significant pathways by a dashed line.

As shown in Figure 8, the direct relationship between long‐term self and episodic memory remained significant when accounting for the common association of age. These results suggest that bidirectional effects may be present. Episodic memory appears to have a stronger influence on the long‐term self (standardized regression weight = 0.50; unstandardized regression weight estimate = 11.25, standard error = 4.96, critical value = 2.27, *p* = .024) than vice versa (standardized regression weight = 0.18; unstandardized regression weight estimate = 0.01, standard error = 0.003, critical value = 2.64, *p* = .008), such that age does not have direct predictive value for the long‐term self in model 1 (standardized regression weight = 0.18; unstandardized regression weight estimate = 0.015, standard error = 0.017, critical value = 0.88, *p* = .378). However, it should be noted that our data are cross‐sectional, and using two unidirectional models to characterize bidirectional mediation effects has been shown through simulation studies to lead to biased estimates of direct and indirect effects. Thus, these weightings should be interpreted with caution (Talluri & Shete, 2018).

## DISCUSSION

Conway and Pleydell‐Pearce's (2000), Conway et al.'s (2004), and Conway's (2005) SMS predicts that self‐specific episodic processing allows for population of the autobiographical memory base, leading to an increase in semantic self‐knowledge, and strengthening the self‐concept as an anchor for incoming autobiographical information (see Figure 1). Our results provide the first developmental support for the SMS, demonstrating that age‐related improvements in self‐specific episodic processing are associated with reciprocal growth in the volume and complexity of self‐knowledge and autobiographical memory across early to late childhood. Following Howe and colleagues' emphasis on the role of self in supporting the development of the mature autobiographical memory system (Howe et al., 2003; Howe & Courage, 1993, 1997, 2004), our findings may also be interpreted as relevant to the gradual offset of childhood amnesia across early to late childhood. Of course, we cannot claim a causal relationship with cross‐sectional, correlational data; and many cognitive (Bauer, 2015), social (Nelson & Fivush, 2020), and neural (Riggins et al., 2020) factors are likely to contribute to the development in autobiographical event processing. Nonetheless, our data might be considered to add empirical weight to the central tenets of SMS theory: age‐related variance in episodic memory is closely related to the development of autobiographical and semantic self‐knowledge.

Replicating the widely cited pattern that has hitherto relied on a small number of isolated studies (Eder, 1989, 1990; Montmayor & Eisen, 1977), we observed an age‐related shift in self‐representation from a focus on concrete self‐knowledge at 3–8 years, to a focus on abstract, psychological self‐knowledge at 9–11 years. This developmental shift might be interpreted as an increase in the complexity and integration of the self‐concept in later childhood, whereby children begin to reflect on their life experiences to abstract more global traits. As predicted, the volume of self‐knowledge also increased with increased life experience, with older children offering more concrete self‐descriptions than the youngest children, and a linear increase in abstract knowledge across early, to middle, to late childhood. The growth in abstract self‐knowledge was not related to children's receptive vocabulary in the current study, although our measure of this factor was affected by an unplanned change of task part‐way through testing, and this knowledge may be related to other untested aspects of language, such as expressive vocabulary and understanding of abstract words (see Cimpian et al., 2017). In the current study, abstract self‐knowledge was best predicted by age and the volume of specific and semantic autobiographical details children provided in their event narratives. In contrast, growth in concrete self‐knowledge was best predicted by specific autobiographical event details. This confirms a close relationship between self‐knowledge and autobiographical knowledge bases, in line with the intuitive reasoning that what we know about ourselves should increase with life experience.

In line with extant research, we also observed a linear age‐related increase in the volume (Hayne et al., 2011) and specificity of detail (Nuttall et al., 2014) provided in children's autobiographical narratives of life events. This is related weakly to children's receptive vocabularies. However, the volume of specific information provided was most strongly predicted by the volume of abstract and concrete self‐knowledge children expressed, and to their success in accurately recalling which actions they had produced in the enactment task. The latter variable was also the strongest predictor of the volume of semantic information provided in event narratives. Importantly, this was a self‐specific relationship, such that children's success in accurately recalling other's actions did not emerge as a strong predictor. This suggests that although we could not assess the accuracy of children's autobiographical event reports, their self‐specific episodic processing capacity, alongside the elaboration of their self‐concept, were key factors predicting age‐related change in the volume of autobiographical event details recalled. This is in keeping with the idea that binding information to autonoetic experience is an important element of autobiographical memorability (Bauer, 2015; Hayne, 2004; Johnson et al., 1993; Newcombe et al., 2000; Perner & Ruffman, 1995; Raj & Bell, 2010; Welch‐Ross, 1995).

The enactment task allowed us to directly assess children's capacity to episodically process their role in a real‐life event, and to bind the information processed within the event to the self‐concept. In keeping with age‐related increases in binding (Sluzenski et al., 2006) and source monitoring ability (Chalmers, 2014; Drummey & Newcombe, 2002; Riggins, 2014), we found age‐related increases in children's ability to recall their own and other's role in recent action events. Moreover, we found an enactment effect: children's memory benefited from autonoetically experiencing an action, as shown by the recall advantage for actions that the child had performed over actions they had witnessed the researcher perform (Badinlou et al., 2017; Ross et al., 2011, 2020). The magnitude of this enactment effect increased with age, such that the effect was more robust in later as compared to early childhood. We predicted an age‐related increase in the enactment effect consistent with a developmental increase in the ability to bind information to the self‐concept, which may gain strength as a mnemonic anchor with increases in volume and complexity in later childhood (see Hutchison et al., 2021). However, recall for own actions was not best predicted by self‐knowledge in the current study, but by age, memory for other's actions, and the strength of the autobiographical memory base. Nonetheless, this was a self‐specific effect; the autobiographical memory base was not predictive of source recall of other's actions, implying that links to the long‐term self may be important only for autonoetic recall.

Our ultimate aim was to explore the role of the SMS in driving the significant quantitative and qualitative change in children's self‐processing and autobiographical memory observed across early to late childhood. Conway and Pleydell‐Pearce's (2000), Conway et al.'s (2004), and Conway's (2005) SMS predicts that episodic processing capacities should be bidirectionally related to the long‐term self, comprised of self‐knowledge and autobiographical memory. SEM suggested that this model offered a good fit to characterize the co‐development of episodic processing, autobiographical memory, and self‐knowledge across 3–11 years. When controlling for measurement error, the relations between self‐knowledge and autobiographical processing were so strong that they were best characterized under one latent variable comprising the “long‐term self,” as described by Conway (2005). It is possible that overlapping task demands contribute to the strong relations between self‐knowledge and autobiographical memory in the current dataset. However, ecologically valid measurement increases the likelihood that findings extend to real‐world event experiences, and the long‐term self was also related to children's episodic processing abilities in the enactment task, which did not rely heavily on verbal ability. This pattern of results strongly supports our novel hypothesis that the *development* of the long‐term self may support—and be supported by—the capacity to recall information about one's own and other's role in real‐world events.

## CONCLUSIONS

Our developmental data enrich the evidence base for the central tenets of SMS theory (Conway, 2005; Conway & Pleydell‐Pearce, 2000; Conway et al., 2004), indicating that children's ability to express knowledge about the self and their past experiences is closely related to their ability to monitor their own and other's roles in events. This pattern can be usefully applied to predict quantitative and qualitative change in children's self‐representations, autobiographical life narratives, and source monitoring ability. Extending extant explanations for childhood amnesia (Howe et al., 2003; Howe & Courage, 1993, 1997, 2004), we suggest that the intertwined development of self and memory across 3–11 years might be usefully applied to understand the protracted early development of our life narrative. Specifically, we propose that together with growth in the capacity to store self‐referent event memories, incremental development in self‐knowledge contributes to the gradual offset of infantile amnesia across childhood. It is unfortunate that despite established developmental change in self in memory across infancy to adolescence (Brummelman & Thomaes, 2017), and the continued prominence of SMS theory (Schacter et al., 2023), relatively few studies have explored the SMS from a developmental perspective. The multidimensional development of self offers natural variance in the capacities captured by the SMS, as applied here to test Conway and Pleydell‐Pearce's (2000), Conway et al.'s (2004), and Conway's (2005) theoretical model. However, development also offers a rich landscape to isolate the contributions of lower‐ and higher‐level self‐processes to memory; from the embodiment that comprises our sense of self in infancy, to the existential self‐consciousness which dominates the adolescent experience, to the fractured sense of self in dementia (see Mentzou & Ross, 2023). Thus, we call for future longitudinal work, applying a developmental perspective to facilitate construction and deconstruction of the SMS, deepening our understanding of the complex interaction between self and memory across the lifespan.

## Data Availability

Materials are available from the first author. The analyses presented here were not pre‐registered. The analytic code is not publicly available due to the mode of analysis, but the data necessary to reproduce the analyses presented here are publicly accessible, at the following URL: https://osf.io/atgvs/.
